# Rare clinical experiences for surgical treatment of melanoma with osseous metastases in Taiwan

**DOI:** 10.1186/1471-2474-8-70

**Published:** 2007-07-25

**Authors:** Kuo-Yuan Huang, Chrong-Reen Wang, Rong-Sen Yang

**Affiliations:** 1 Department of Orthopaedics and Institute of Clinical Medicine, College of Medicine, National Cheng Kung University Medical Center, Tainan city, Taiwan; 2 Section of Rheumatology and Immunology, Department of Internal Medicine, College of Medicine, National Cheng Kung University Medical Center, Tainan city, Taiwan; 3 Department of Orthopaedics, College of Medicine, National Taiwan University Medical Center, Taipei city, Taiwan

## Abstract

**Background:**

Malignant melanoma occurs infrequently in Taiwan. Once it has progressed into osseous metastases, the prognosis is poor. There are no reported clinical experiences of surgical management in this area.

**Methods:**

To improve our understanding of the rare clinical experiences, we retrospectively investigated clinical characteristics, radiological findings, treatment modalities, survival outcomes and prognoses of 11 Taiwanese patients with osseous metastasis of melanoma treated surgically at two national medical centers, National Taiwan University Hospital and National Cheng Kung University Hospital from January 1983 to December 2006.

**Results:**

Six patients suffered from acral-lentiginous melanoma. Nine patients sustained multiple osseous metastases and most lesions were osteolytic. Nine patients also had sustained metastases to other organs including liver, lungs, lymph nodes, brain and spleen. Second malignancies including lung cancer, thyroid papillary carcinoma, renal cell carcinoma and cervical cancer co-existed in four patients. The interval from the initial diagnosis of melanoma to the clinical detection of osseous metastases varied from 0–37.8 months (mean 9.75 months). Metastatic melanoma was invariably fatal; the mean survival time from bone metastases to death was 5.67 months.

**Conclusion:**

Due to the high morbidity and poor survival of Taiwanese patients with osseous metastases of melanoma, surgical treatment should be directed towards pain relief and the prevention of skeletal debilitation in order to maintain their quality of life.

## Background

After melanoma has grown radially and superficially in the epidermis and on mucosal surfaces, it can metastasize to any organ or tissue such as the lungs and bone, where melanoma metastases are common [1,2]. Melanoma most frequently occurs in fair-skinned Caucasian individuals older than 40 years who have numerous moles [3]. In most countries, the incidence of melanoma has been increasing recently, with annual incidences reaching 3–7% among fair-skinned populations [3,4]. In the United States, the incidence of melanoma has increased more than that of any other cancer [5]. An estimated 60,000 new cases of melanoma are diagnosed each year with more than 8,000 annual deaths in the United States; about one in 75 persons will develop this cancer in their lifetime [5,6]. After osseous metastases occur, the patient's survival is usually short-term [7,8]. In addition, bone metastases of melanoma predispose patients to pathological fractures of axial and appendicular skeletons, bone marrow failure and neurological deficits. These complications substantially increase their pain and result in a poor quality of life [6,7,9].

Despite its high mortality rate, melanoma is not well studied in Asians, with only a limited number of reported cases [10]. Acral-lentiginous melanoma (ALM) is the most common subtype of melanoma in Asian populations including Taiwanese [11]. The diagnosis of this subtype is often delayed [11,12]. We conducted this retrospective chart review in two national medical centers in Taiwan to improve our understanding of the incidence, treatment and outcomes of osseous metastases of melanoma in Asia.

## Methods

The hospital records of the National Taiwan University Hospital and the National Cheng Kung University Hospital from January 1983 to December 2006 were surveyed to identify cases of malignant melanoma. We reviewed the charts, medical records, surgical notes, radiological images and pathology records of patients diagnosed with osseous metastases. A total of 11 patients underwent surgical intervention for osseous metastases. Surgical indications in our series included an intractable skin ulcer, mechanical instability due to pathologic fractures, neurological deterioration due to neural compression by metastases, pain unresponsive to nonoperative measures such as radiation and the need for biopsy material for histopathological verification of the diagnosis. The choice of surgical procedure depended on the patients' individual clinical situation, location of the tumor, medical comorbidities and overall prognosis. Surgical treatments included wide excision (four patients), posterior laminectomy for decompression and posterior instrumentation (one patient), below-the-knee amputation (two patients), above-the-knee amputation (one patient), ray amputation (one patient) and intralesional excision plus internal fixation with cement augmentation (two patients) (Tables 1 &2).

**Table 1:** Clinical, pathological and radiographic data

Patient age (years)	Sex	Primary tumor	Clinical size (cm)	Microscopic classification	Depth (Clark level)	Osseous metastases and complications	Radiographic manifestation	Other metastases	Synchronous tumor
45	F	Scalp	1.5 × 1.0	Malignant melanoma	V	Frontoparietal skull, multiple cervico- thoracolumbar spine (compression fractures of thoracolumbar spine), bone marrow infiltration	Multiple spinal metastases with bony destruction and cord compression	Liver, bone marrow	None
72	M	L anterior chest wall	1.5 × 1.5	Malignant melanoma	IV	5^th ^lumbar vertebra, R acetabulum	L5 compression fracture with bony destruction	Lung, Lymph nodes	None
64	M	R sole	1.5 × 1.5	acral lentiginous melanoma	V	L skull base, midcervical, mid thoracolumbar spine	Multiple bony destruction, permeative osteolytic skull lesion	Lung	None
79	M	L sole	1.0 × 1.0	acral lentiginous melanoma	III	R scapular, thoracolumbar spine (L2 compression fracture), L sacroiliac joint, pelvic rim and bilateral femurs	Multiple bony destruction with periosteal reaction and dystrophic calcifications	Lymph nodes	Lung cancer
62	M	L sole	2.3 × 2.1	acral lentiginous melanoma	IV	L ninth rib, thoracolumbar spine	Sclerotic lesions in thoracolumbar spine	Liver	Thyroid papillary carcinoma, renal cell carcinoma
67	F	R heel	3.5 × 1.0	acral lentiginous melanoma	V	R femoral pathological fracture, R knee, L mid femur, R scapular, rib	Permeative osteolytic change	Lymph node, lung, liver	None
70	F	L lower leg	1.0 × 1.0	Malignant melanoma	IV	L femoral subtrochanteric fracture	Osteolytic bony destruction	Liver, spleen	Renal cell carcinoma
67	M	R foot	7.0 × 6.0	acral lentiginous melanoma	V	R tibia, proximal femur	Increased tracer uptake over R proximal femur	Lung, lymph nodes	None
39	F	L subungual	1.1 × 0.9	acral lentiginous melanoma	V	Skull, thoracolumbar spine, thoracic cage, pelvic rim	Osteolytic bony destruction	Liver, lung, lymph nodes, brain	None
74	F	L lower limb	1.0 × 0.8	Malignant melanoma	V	L midtibial pathological fracture, sacroiliac joint	Osteolytic bony destruction	Lung	Cervical cancer
51	M	Unknown	NA	Malignant melanoma	NA	R navicular pathological fracture	Comminuted fracture	Liver, lung, lymph nodes	None

L, left; R, right; NA, not applicable.

**Table 2:** Surgical treatments and outcomes

Patient age (years)	Sex	Surgical treatment	Therapy	Time (months)		
				
				Diagnosis to bone metastases	Diagnosis to death	Bone metastases to death
45	F	Wide excision	Radiotherapy	1.77	7.44	5.67
72	M	Posterior laminectomy for decompression, vertebroplasty with PMMA cement, posterior instrumentation	Radiotherapy, chemotherapy, IL-2	16	17.27	1.27
64	M	Below-knee amputation	Radiotherapy, BCG, chemotherapy	14.00	18.27	4.27
79	M	Wide excision, transposition flap, lymph node dissection	Radiotherapy	15.90	21.87	5.97
62	M	Wide excision	None	0.70	16.40	15.70
67	F	Wide excision, groin dissection, ORIF with Küntscher nailing and cement augmentation	Radiotherapy	37.80	38.97	1.17
70	F	ORIF with CHS and bone cement augmentation	Chemotherapy	8.87	13.67	4.80
67	M	Below-knee amputation	Chemotherapy	3.57	7.77	4.20
39	F	Ray amputation	Interferon and tamoxifen	6.20	7.30	1.10
74	F	Above-knee amputation	Chemotherapy	2.43	10.76	8.33
51	M	None*	Chemotherapy	0.00	9.87	9.87

BCG, Bacillus Calmette-Guérin; ORIF, open reduction and internal fixation.

*Diagnosed with fine-needle biopsy.

All patients had histopathological proof of bone metastases. Table 1 lists the locations of their osseous metastases, and Table 2 shows the operations performed. Adjuvant therapies after surgical treatment varied depending on the patient's age, the sites and number of metastases, disease progression, the patient's overall medical condition and the patient's intentions for further management. Table 2 lists the adjuvant therapies including chemotherapy with or without tamoxifen (six patients), radiotherapy (five patients) and immunotherapy with Bacillus Calmette-Guérin (BCG), interferon alpha or IL-2 (three patients).

We compiled data on the age and sex distribution of these patients, their primary lesions, clinical sizes, microscopic classification with depth of invasion (Clark level), bone metastases and complications, radiographic manifestations, other metastases, synchronous tumors, usages of analgesic medications, interval from initial diagnosis to occurrence of osseous metastases, interval from diagnosis to death and survival after the occurrence of bone metastases.

## Results

**Figure 1:**
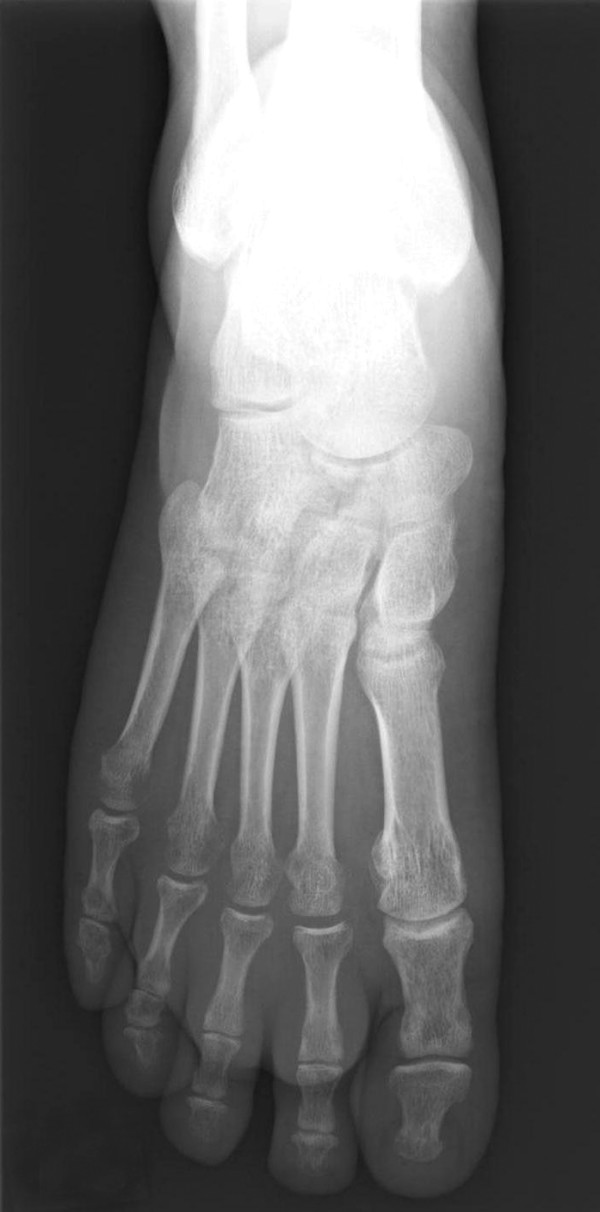
Pathologic fracture of the right naviculum in a 51-year-old man with melanoma presented at the initial visit.

**Figure 2:**
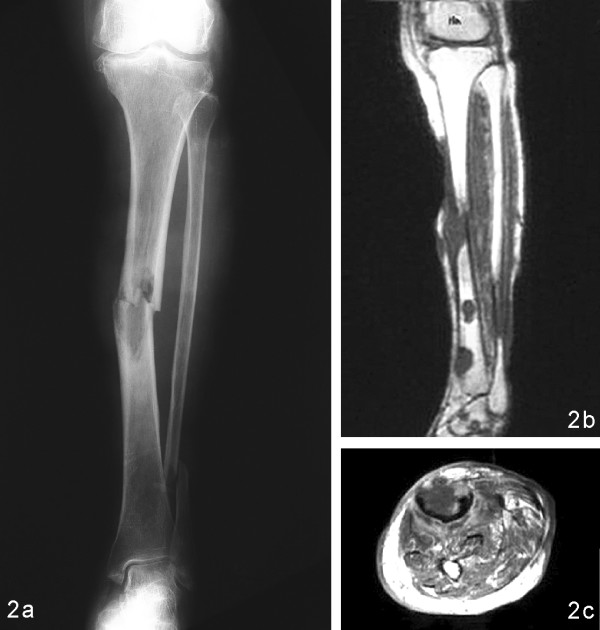
**(a)**. Plain radiograph of a 74-year-old woman. The radiograph reveals a pathologic fracture at the diaphysis of the left tibia due to an osteolytic lesion in the medullary cavity. **(b)**. T1-weighted magnetic resonance image showing several tibial lesions that are eccentric, oval and associated with clinically significant cortical erosion. **(c)**. T1-weighted magnetic resonance image demonstrating a hypointense lesion with endosteal scalloping of the tibia and cortical erosion.

The 11 patients (six men and five women) ranged in age from 39–79 years (mean 61.8 years) at the time of diagnosis of melanoma. Six patients had the ALM subtype. The metastases of the melanoma were correlated with the severity of the primary cutaneous lesions (Table 1). One patient who did not possess a primary cutaneous lesion had a melanoma of the navicular bone (Fig. 1), with an initial presentation of dull pain in the right midfoot that lasted six months. Most patients had pain and weakness in the involved regions. Osteolytic bone lesions were radiographically detected in 10 patients. Osteosclerosis of the thoracolumbar spine was also found (Table 1). Most lesions in the long bones were multiple, eccentric and oval, with clinically significant cortical erosion (Fig. 2). Two patients had permeated osteolytic lesions; such a pattern was characterized by numerous, tiny radiolucencies between the trabeculae of the residual bones. Periosteal reactions and dystrophic calcifications were found in one patient with multiple sites of bony destruction in the ileum and sacrum.

**Figure 3:**
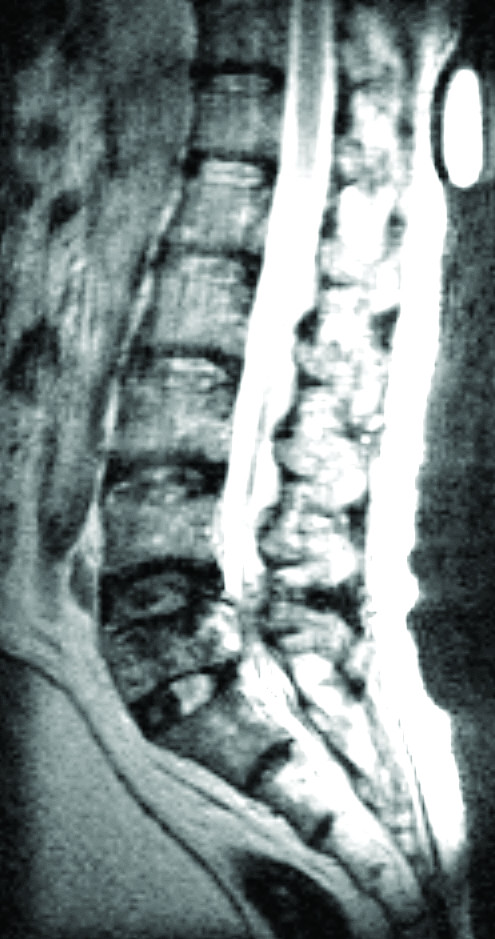
Magnetic resonance image of lumbosacral spine showing a compression fracture of L5 of posterior bulging contour with enhancement and epidural soft tissue mass over the anterior epidural space with marked compression of the thecal sac.

The osseous metastases were in the axial skeleton in eight patients, six of whom had spinal metastases. Two patients had osseous metastases to the cervical spine, four to the thoracic spine, six to the lumbar spine, two to the scapula, three to the ribs, four to the pelvis and three to the skull. Compression fractures with epidural compression occurred in three patients with spinal metastases. One patient received posterior laminectomy for decompression and posterior instrumentation because of progressive muscle power weakness of right lower limb (Fig. 3). The other two patients received irradiation instead of laminectomy because of their poor medical condition. The appendicular skeletons were involved in six patients, with bone metastases to the femur in four patients, to the tibia in two and to the navicular bone in one. Four patients had pathological fractures including one each in the subtrochanteric region of the femur, the midshaft of the femur, the midshaft of the tibia, and the navicular bone. Two patients with femoral fractures were successfully treated with intralesional excision and internal fixation with compression hip screws and Küntscher nailing augmented with cement fixation. The patient with the tibia fracture was treated with above-the-knee amputation because of multiple skin lesions and ulcers. The patient with the navicular fracture underwent conservative treatment with chemotherapy alone because of the coexistence of several distant metastases.

All 11 patients had metastases other than osseous metastases from their melanomas. Four patients had a synchronous malignancy including one lung cancer, one thyroid papillary carcinoma, two renal cell carcinomas and one cervical cancer. The synchronous malignancy usually occurred first and then co-existed with the melanoma (Table 1). The use of analgesic medications was decreased after the operations, and most patients reported significant alleviation of pain after surgical intervention. Dramatic pain relief was achieved in three patients with pathological fractures who received internal fixation.

The interval from the diagnosis of melanoma to the detection of osseous metastases varied from 0–37.8 months (mean 9.75 months) (Table 2). The prognosis of patients with melanoma and osseous metastases was generally poor. After osseous metastasis had been diagnosed, the mean survival was only 5.67 months (Table 2). We found no significant differences in prognosis between the axial and appendicular osseous metastases (mean survival 5.44 and5.72 months, respectively). The average survival time of males (6.88 months) was longer than females (4.21 months). There was no obvious correlation between age and survival. The survival time of six patients with the ALM subtype averaged 5.40 months, which was only slightly shorter than that of the other five patients (5.99 months). Even in patients with a synchronous tumor, the prognosis did not deteriorate. There were no distinct differences in survival among patients receiving chemotherapy, radiotherapy and/or immunotherapy.

## Discussion

Osseous metastases of malignant melanoma are rare among Asians [10,11]. Six of our patients had ALM, which has the predilection to occur on the palms, soles and subungual nail beds. Such a distribution usually delays diagnosis and patients accordingly have worsened prognoses. Indeed, presently we found that the survival time of ALM patients (5.4 months) was slightly shorter than that of other subtypes (5.99 months). Osseous metastases from malignant melanoma usually occur in patients with relatively advanced primary lesions and multiple bony lesions at the time of diagnosis [12-14], as shown in the histopathological findings of our series with a Clark level of III, IV or V. Until now, there has not been evidence to support the conclusion that the surgical treatment contributes to the survival of metastatic melanoma patients [1,8,15]. Therefore, management is directed towards the relief of symptoms and the maintenance of a good quality of life in patients with osseous metastases of melanoma.

Primary melanoma of the bone has been rarely reported in the literature [16]. In isolated appendicular osseous metastases of melanoma, survival may be prolonged after aggressive surgical resection of the metastatic bony focus [17]. Common sites for osseous metastases of melanoma include the vertebrae, skull, pelvis, thoracic cage and proximal parts of the femur [18-22]. Osseous metastases generally occur in the axial skeleton; reflecting this, there were eight cases among the 11 patients presently studied. Spinal metastases may result in vertebral compression fractures, epidural or cord compression (Fig. 3) [6,7,9]. For patients with neurological deficits such as paraplegia or cauda equina syndrome, the neurological decompression to relieve symptoms and recover functions should be considered as health permits. Moreover, simultaneous instrumentation or vertebroplasty with polymethylmethacrylate (PMMA) cement can provide spinal stability to alleviate back pain in patients with pathological vertebral fractures. Intralesional excision or curettage of osseous metastatic melanoma lesions together with open reduction and internal fixation with PMMA cement augmentation might be an effective strategy for the surgical management of pathological fractures in the appendicular skeleton. Two patients in our series received such an intervention and obtained significant pain relief. Metastatic lesions within the long bones are usually multiple, osteolytic, eccentric and oval, and are associated with clinically significant cortical erosion [15]; this was evident in our cases (Fig. 2). Lesions usually appear first in the medullary cavity, and then spread to destroy the bone trabeculae, extend to the cortex and finally lead to pathological fractures (Fig. 2) [14,15]. However, such radiographic features in our patients were similar to those in other cancers.

Because melanoma with osseous metastases is heterogeneous in its biology, treatment plans must be highly individualized. Surgical management is generally followed by adjuvant therapies such as chemotherapy, radiation therapy or immunotherapy. Single or combined chemotherapy does not improve survival in advanced-stage disease, as evident presently and reported previously [1,8,10]. External beam radiotherapy is effective in preventing local recurrence of malignant melanoma and can provide palliative treatment for metastatic melanoma [23]. In our cases, irradiation of the affected areas relieved pain considerably for several months without affecting the growth of tumor, similar to previous reports [10,15]. Significant proportions of melanoma cells express estrogen receptors, which leds to a supportive role for tamoxifen in the treatment of metastatic melanoma [8]. Given their immunologic effects, low-dose interleukin-2, granulocyte-monocyte colony stimulating factor and interferon alpha-2b produce durable remissions in patients with metastatic melanoma, and they have been used as postoperative adjuvant therapies [24]. Therefore, the goal of surgical treatment for osseous metastases of melanoma should be directed towards the palliation of symptoms, particularly if the anticipated relief of related symptoms exceeds the potential adverse effects of the therapy. Symptomatic metastases in weight-bearing bones require special consideration. For large lesions or cortical destruction, prophylactic stabilization and irradiation are preferred. Alternatively, the lesion may be treated with radiation alone and patients are monitored for evidence of pathologic fractures. Unless the risk of surgery is high or the expected life span is short, pathologic fractures of weight-bearing bones should be stabilized to maximize the quality of life and decrease hospital costs. Furthermore, surgical resection can produce an immediate decrease in tumor burden at a reasonable expenditure [25].

Bisphosphonates such as pamidronate and zoledronic acid have been used for palliative therapy in patients with osteolytic lesions metastasized from multiple myeloma or metastatic breast cancer. The aim is to prevent or delay the onset of skeletal complications [26,27]. Moreover, bisphosphonates can reduce bone pain in patients with bony metastases [28]. Zoledronic acid has demonstrated efficacy in the treatment of bone metastases in patients with prostate cancer, lung cancer or other solid tumors [26]. The bisphosphonate pamidronate induces apoptosis in human melanoma cells in vitro [29]. In an in vivo animal study, incadronate inhibited bone resorption by increasing numbers of apoptotic osteoclasts [30]. In addition, incadronate can substantially suppress the growth of human melanoma cells, increase the numbers of apoptosis of tumor cells and decrease the tumor-associated blood-vessel density [30]. Therefore, bisphosphonate therapy might be an alternative to surgery or an adjuvant treatment for osseous metastatic melanoma in the future.

The prognosis of patients with melanoma is related to the extent and the stage of the tumor. Early detection and management of melanoma are the key factors to improve overall survival. The prognosis is poor after melanoma progresses and undergoes osseous metastasis [2,31]. Osseous metastases of melanoma may predispose individuals to pathologic fractures in the axial or appendicular bones or to bone marrow failure and may substantially influence the quality of life and survival expectancy of patients. For Asian patients with melanoma, the mean survival after the diagnosis of osseous metastases was 5.67 months in our series, which was longer than 4.7 months reported in Caucasian populations [2,7,31].

## Conclusion

In conclusion, osseous metastases of melanoma are rare in Taiwan and the prognoses of patients are as poor as with Caucasians. Surgical intervention is effective in providing pain relief, especially for those with pathological fracture and those receiving internal fixation. Due to the high morbidity and poor survival, surgical treatment should be directed towards pain relief and the prevention of skeletal debilitation in order to maintain a patient's quality of life.

## Competing interests

The author(s) declare that they have no competing interests.

## Authors' contributions

KYH conceived the study, participated in its design, collected the data, and drafting of the manuscript.

CRW did the data analysis, and revised the manuscript.

RSY assisted in the creation and design of the study, collected the data, advised and assisted drafting of the manuscript.

All authors read and approved the final manuscript.

## Pre-publication history

The pre-publication history for this paper can be accessed here:


